# Baldness

**Published:** 1891-02

**Authors:** 


### BALDNESS.

Dyspepsia is one of the most common causes of baldness. Nature is a great economizer, and when the nutrient elements furnished by the blood are insufficient to properly support the whole body, she cuts off the supply to parts the least vital, like the hair and nails, that the heart, lungs and other vital organs may be the better nourished. In cases of severe fevers, this economy is particularly noticeable. A single hair is a sort of history of the physical condition of an individual during the time it has been growing, if one could read closely enough. Take a hair from the beard or from the head and scrutinize it, and you will see that it shows some attenuated places, indicating that at some period of its growth the blood supply was deficient from overwork, anxiety, or under feeding. The hair falls out when the strength of its roots is insufficient to sustain its weight any longer, and a new hair will take its place unless the root is diseased. For this reason, each person has a certain definite length of hair. When the hair begins to split or fall out, massage to the scalp is excellent. Place the tip of the fingers firmly upon the scalp and then vibrate or move the scalp while holding the pressure steadily. This will stimulate the blood vessels underneath and bring about better nourishment of the hair. A brush of unevenly tufted bristles is also excellent to use upon the scalp, not the hair.

